# Sustainability Standards in Pediatric Anesthesia: Quality Initiative to Reduce Costly Environmentally Harmful Volatile Anesthetics

**DOI:** 10.1097/pq9.0000000000000708

**Published:** 2023-12-12

**Authors:** Andrew T. Waberski, Sophie R. Pestieau, Caroll Vazquez-Colon, Jessica Cronin, Barbara H. Braffett

**Affiliations:** From the * George Washington School of Medicine, Department of Anesthesiology, Pain, and Perioperative Medicine, Children’s National Hospital, Washington, D.C.; † Department of Epidemiology, Milken Institute School of Public Health, The Biostatistics Center, George Washington University, Washington, D.C.

## Abstract

**Background:**

The emission and entrapment of greenhouse gases (GHG) inside the atmosphere is one of the leading causes of global warming. Commonly administered anesthetics have global warming potential up to 2,000 times greater than carbon dioxide. This Quality Improvement (QI) initiative aimed to develop a set of sustainability standards to reduce volatile anesthetic GHG emissions and costs at a children’s hospital.

**Methods:**

In January 2020, the QI project team implemented education sessions for clinical staff on the environmental impact of volatile anesthetics, bedside clinical reminders, resource guides on sustainable anesthesia practices, preset low-flow gas levels on anesthesia machines, relocated and reduced the number of available vaporizers, and implemented policies to standardize clinical practice. Using hospital pharmacy purchase order data between 2018 and 2022, GHG emissions and costs from three commonly used volatile anesthetics (Isoflurane, Sevoflurane, and Desflurane) were compared using metric ton carbon dioxide equivalents.

**Results:**

During 3 years, GHG emissions from volatile anesthetics were significantly reduced by 77%, with most of the reduction attributed to the reduced use and eventual elimination of Desflurane. Purchase costs were also significantly reduced during this period by 41%.

**Conclusions:**

This QI project successfully decreased GHG emissions over 3 years by simultaneously reducing the use of costly and environmentally harmful volatile anesthetic, Desflurane, and increasing the use of low-flow anesthesia. This study addresses our anesthesia practices and healthcare system’s impact on the pediatric population and proposes simple interventions to mitigate the negative consequences of current practices.

## INTRODUCTION

### Problem Description

Healthcare systems are designed to preserve and improve the well-being of humans, while sustainable practices aim at conserving the natural world through efficient, ethical, and fiscally responsible choices. Together, healthcare systems are directly responsible for sustainable practice by protecting both humans and the planet. Healthcare is responsible for 10% of US greenhouse gas (GHG) emissions.^1^ Healthcare-related GHG emissions typically originate from supply-chain, purchased energy, and healthcare facilities.^2^ Within the operating room, anesthetic gases and volatile agents contribute to more than half of the GHG emissions.^3^

### Available Knowledge

Desflurane has the highest radiative efficiency and longest atmospheric lifetime among volatile gases routinely used in clinical practice (Desflurane, Sevoflurane, Isoflurane, and Nitrous Oxide).^4,5^ The environmental impact is typically expressed as global warming potential (GWP) and compares a gas’s 100-year global warming contribution relative to carbon dioxide CO_2_ (GWP of 1). Compared with other volatile anesthetics, Desflurane has 15–20 times the life cycle GHG emissions for resource extraction, drug manufacturing, transport to facilities, delivery to patients, and disposal.^6^ In addition, Desflurane is the costliest volatile anesthetic; it is two times as expensive as Sevoflurane and up to four times when compared with Isoflurane.^7^

### Specific Aim

The global aim of this Quality Improvement (QI) initiative was to develop a multipart, clinically suitable set of sustainability standards aimed at reducing GHG emissions of volatile anesthetics, particularly Desflurane, and to secondarily decrease the overhead cost of volatile anesthetics at a tertiary-care pediatric hospital. Our primary aim was to reduce CO_2_ emission from volatile anesthetic use in all anesthetizing locations at Children’s National Hospital (estimated to be 156 metric tons per quarter) by 50% and to sustain the reduction for 6 months.

## METHODS

### Context

This QI initiative was developed and implemented at Children’s National Hospital (CNH), a nonprofit academic tertiary-care pediatric hospital in the Washington, DC metropolitan area. All anesthesia services are supervised by subspecialty-trained pediatric anesthesiologists providing solitary care or care in conjunction with an anesthesia provider (eg, anesthesia assistants, residents, or fellows). The pediatric patient population at Children’s National includes a broad age spectrum of patients as young as newborns and infants, up to 22 years of age, and occasionally includes adult patients with congenital disease. The clinician decides the safest dose and choice of volatile anesthetic based on the patient’s clinical status and comorbidities. Volatile anesthetics are used extensively in anesthesia services throughout various sites at Children’s National, including radiology, cardiac catheterization, hematology-oncology procedures, and many operative services.

### Interventions

**Fig. 1.**
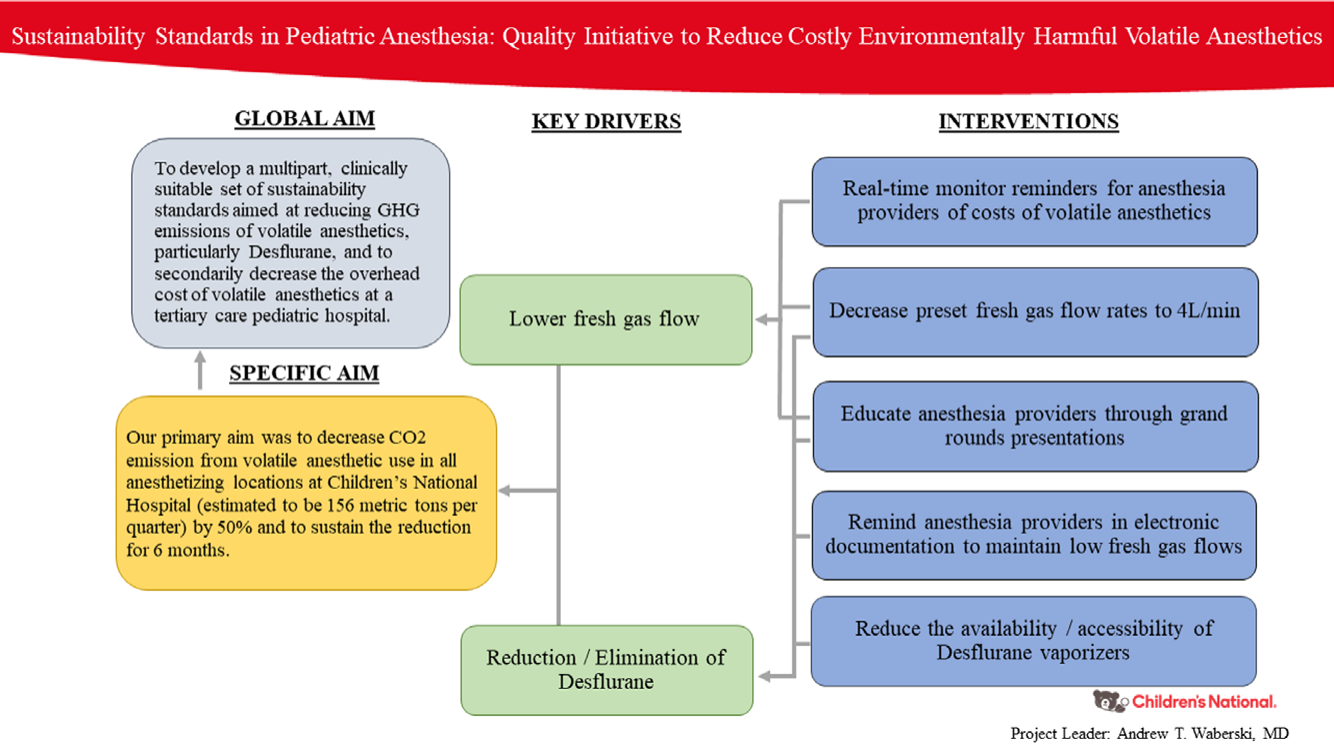
Key driver diagram.

The QI project team consisted of 2 pediatric anesthesiologists, with appointments to the hospital-wide committee focused on sustainability (CNH Sustainability Council), nine pediatric anesthesia fellows, a clinical pharmacist, a supply-chain representative, and the hospital’s lead anesthesia technician. The membership of the QI team was critical to ensure that key stakeholders were present and engaged with initiating and implementing the QI initiative. The QI team also solicited information and guidance from the Society for Pediatric Anesthesia Sustainability Special Interest Group. In November 2019, the QI team reviewed hospital pharmacy purchase order data spanning Q3 of 2018 through Q3 of 2019 as part of a current state analysis to determine the recent usage of volatile anesthetics at Children’s National. Concurrently, the QI project team met monthly to examine current practices and guidelines and to develop new standards for environmentally sustainable and lower-cost anesthesia practice. We developed a key driver diagram outlining the primary drivers and proposed interventions (Fig. 1). Interventions included: anesthesia provider education, decreased preset fresh gas flow settings on anesthesia ventilators at the beginning of each anesthesia case, electronic reminders for anesthesia providers to reduce fresh gas flows in the electronic medical record, real-time displays of costs associated with volatile anesthesia use on the anesthesia machine, and reduced accessibility of Desflurane vaporizers. The anesthesia delivery machines modified at Children’s National Hospital during this study were the Aisys CS2 machine and Carestation 650 by GE Healthcare. The electronic medical record modified was the Cerner SurgiNet: Anesthesia program. Every intervention was performed in in January 2020 at all available anesthetizing locations.

#### Anesthesia Provider Education

We educated providers in a common, well-attended, and professional Grand Rounds presentation setting. This format of lecture followed by a question-and-answer session was used to include and motivate providers to participate in the project through discussions that resolve uncertainties, spread practice information, and gain trust by exposure. Resource guides on best practice standards were shared by email with each provider and acted as a source of assimilated information with tools for direct application to clinical practice and shared teaching. We equipped each clinical workstation with a detailed resource guide on the environmental impacts of different volatile anesthetics at different flow rates and sustainable anesthetic techniques. The team implemented a clinical resource guide to maintain project visibility, be a source of bedside discussion for providers, and inform new rotating trainees of the implemented QI project. In addition, new residents were informed about the sustainability initiative as part of their orientation to the hospital.

#### Decreased Preset Fresh Gas Flow Settings on Anesthesia Ventilators

The QI team leveraged other tools beyond education to support a sustained shift in anesthesia provider behavior. The team-modified anesthesia ventilator presets to administer lower, yet clinically relevant, gas flow rates (4 L/min) at the beginning of each anesthesia case. This systemic change created an “opt-out” model so that the default was set to low gas flow; anesthesia providers could still change the settings as needed. The QI team also created “hotkeys” (quick access on-screen buttons) on the anesthesia ventilators for the maintenance of gas flow levels (0.5 L/min and 1 L/min), which made it easier for anesthesia providers to consistently use low gas flows, when possible.

#### Electronic Reminders in the Electronic Medical Record

Within the electronic medical records, the team created interactive cues for each anesthesia case to encourage participation in reducing anesthetic waste. These cues seemed as a workflow tab labeled “Use low fresh gas flow” in the electronic medical chart for every patient undergoing anesthesia.

#### Real-Time Displays of Costs Associated with Volatile Anesthesia Use

The QI team changed the default anesthesia workstation screen to display a real-time cost per hour of designated volatile anesthetics. This clinically relevant immediate feedback provided positive reinforcement to reduce costs by decreasing unnecessary volatile anesthetic use due to high gas flows.

#### Reduced Accessibility of Desflurane Vaporizers

Before this QI project, there was a Desflurane vaporizer in every anesthetizing location at Children’s National. With the support of the anesthesia technicians, the QI team moved these vaporizers to anesthesia equipment storage areas adjacent to the clinical areas. The QI team also reduced the number of available vaporizers from 18 to 3. Anesthesia providers could still access Desflurane vaporizers, if needed.

### Measures

The primary aim was to decrease CO_2_ emission from volatile anesthetic use in all anesthetizing locations at Children’s National by 50% and to sustain the reduction for 6 months. At Children’s National, volatile anesthetics are purchased through the pharmacy by anesthesia technicians every 2 weeks, and drug purchase histories, including the type of volatile anesthetic, purchase date, purchase price, and volume of anesthetic, are documented by the pharmacy purchasing supervisor. Using hospital pharmacy purchase order data spanning Q3 of 2018 through Q2 of 2022, GHG emissions from three clinically used volatile anesthetics (Isoflurane, Sevoflurane, and Desflurane) were compared using metric ton CO_2_ equivalents (MTCO_2_e). The specific gravity of each anesthetic was used to convert the calculated case volume of liquid anesthetic to weight in kilograms. Calculations of MTCO_2_e were made by multiplying the kilogram weight of the anesthetic by the associated 100-year GWP of the anesthetic, and converting it to metric tons.^8–10^ Calculated emissions of 1 kg of Desflurane and Isoflurane, respectively, are equivalent to emissions of 2,540 and 510 kg of CO_2_. Alternatively, 1 kg of Desflurane contributes 2,540 times more global warming over 100 years compared with 1 kilogram of CO_2_.

### Analysis

We plotted the outcome measures on statistical process control U-charts and X bar S charts.

### Ethical Considerations

This project was considered a QI initiative at Children’s National and, as such, did not constitute human subjects research and did not require oversight by the institutional review board. Individual patient and provider care and outcomes were not assessed in relation to these interventions. No changes to the goals and expectations of care were implemented.

## RESULTS

At the time of implementation in January 2020, the anesthesiology division included 47 attending anesthesiologists, 10 pediatric fellow anesthesiologists, 11 anesthesia assistants, and 5 residents rotating from outside institutions.

**Fig. 2.**
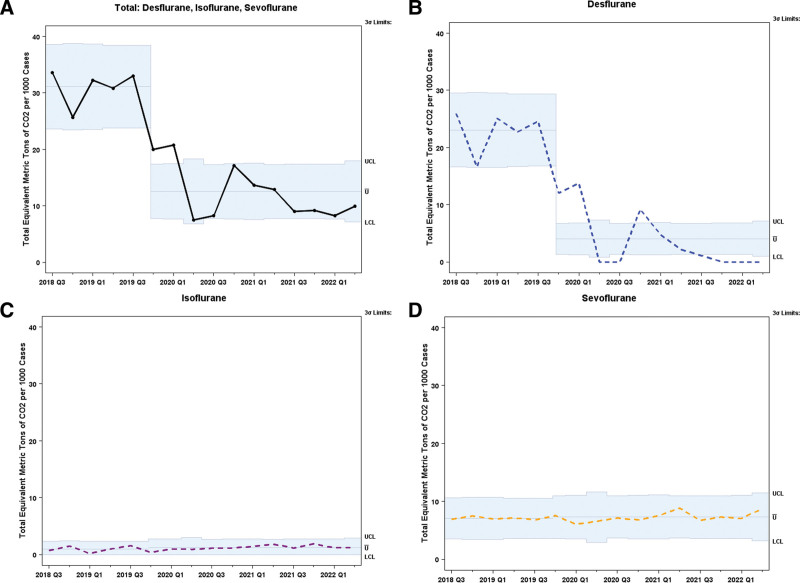
u-Chart for equivalent metric tons of CO_2_ per 1000 Cases.

There was a significant reduction in GHG emissions during the period before the implementation of the sustainability standards (Q3 2018–Q3 2019) to after implementation (Q4 2019–Q2 2022) (Fig. 2). GHG emissions from three important volatile anesthetics (Isoflurane, Sevoflurane, and Desflurane) peaked in Q3 2019 at a combined total of 172 MTCO_2_e, whereas more recent results from Q2 2022 demonstrated a low of 38 MTCO_2_e. The number of cases remained constant between 2018 and 2022 (~4500 cases), except for a 25% decrease in Q2 2020 due to the COVID-19 pandemic.

**Fig. 3.**
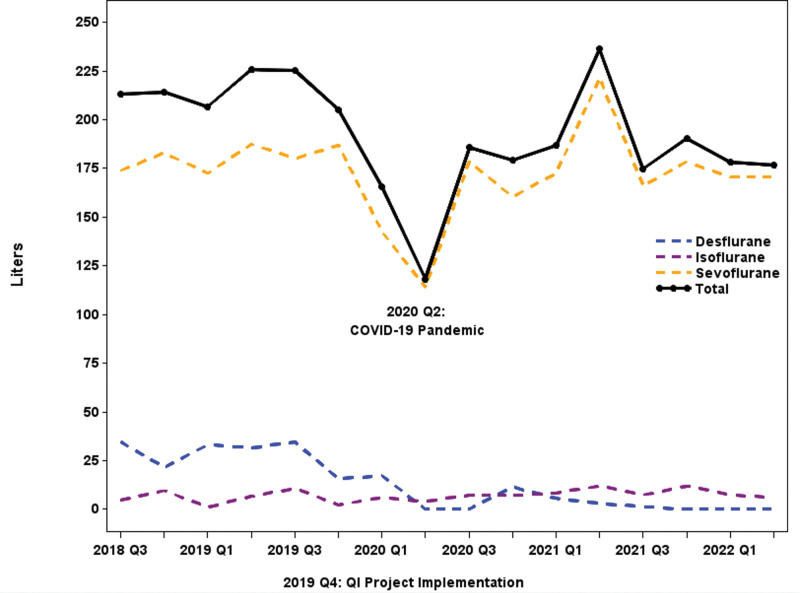
Liters of volatile anesthetic used per quarter.

Most of the reduction in MTCO_2_e came from the reduced use and eventual elimination of Desflurane from clinical practice, which has an exceedingly high accompanying 100-year GWP (Fig. 2). The total volume of volatile anesthetics used decreased from 213L in Q3 if 2018 to 177L in Q2 2022 (Fig. 3). In 2018, Desflurane represented 16% of the total anesthetic volume and 77% of the total MTCO_2_e. By 2022, Desflurane was eliminated from practice at Children’s National. Additionally, reductions in Desflurane were not significantly compensated for by excessive use of Sevoflurane or Isoflurane; volumes remained constant in the later quarters as Desflurane phased out (Fig. 3).

**Fig. 4.**
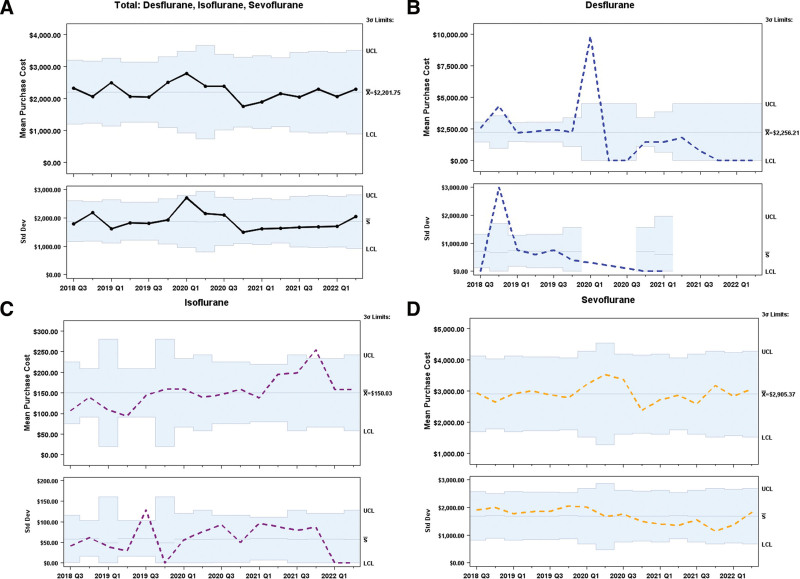
X bar S chart for purchase cost of volatile anesthetics per quarter.

Average purchase costs per quarter were also significantly reduced during these 3 years (**Fig. 4**, $2.59K Q3 2018 versus $0.74K Q1 2021). Desflurane represented 28% of the total volatile anesthetic purchase cost. With the significant reductions in Desflurane, our contractual obligations with the pharmaceutical companies required a minimum number of Desflurane bottles to be ordered annually. By October 2021, our department had consumed all of the remaining Desflurane, and as a result, we removed all Desflurane vaporizers by February 2022.

## DISCUSSION

### Summary

Successful implementation of the QI initiative to reduce GWP of volatile anesthetics in a tertiary pediatric facility resulted in significant reductions in GHG emissions during a 3-year period. CO_2_ emission from volatile anesthetic use in all anesthetizing locations decreased from 172 MTCO_2_e at its peak in Q3 2019 to a low of 38 MTCO_2_e in Q2 2022. Desflurane use decreased from 35L in Q3 2018 to 0L in Q2 2022. Even with Desflurane’s reduction and eventual elimination, significant decreases were still observed in the total volume of volatile anesthetics used, indicating anesthesia providers were using low-flow techniques. As a result, purchase costs were reduced by 41%, a cost savings of more than half a million dollars over 10 years.

### Interpretation

In January 2020, our team implemented two key drivers, including decreasing fresh gas flow and reducing the use of Desflurane. The observed reductions in emissions and costs were accomplished by indirect and direct interventions to elicit the most provider engagement, clinical response, and adherence. Indirect interventions for provider engagement included systems-based policies and were reinforced by continued education on environmental impact and sustainability. Direct interventions aimed to reduce burdensome tasks for providers, provide positive reinforcement for sustainable practice, and remove access to less sustainable choices. This QI initiative was successful due in part to the multiple levels of engagement of participating leaders, staff, and educators. This team-based approach, in addition to the continued educational resources and leadership support, resulted in an effective and maintainable QI initiative.

Future areas of study could focus on total intravenous anesthetic techniques and reducing the use of other environmentally harmful gas anesthetics, including Nitrous Oxide.^11^

### Limitations

Limitations to the QI project involved a paucity of provider-based evaluations of the project’s impact on clinical practice, workflow, and preferences. Generalizability to other institutions may be limited based on the resources and personnel available at other institutions. Tools deployed by our QI team (use of electronic medical records reminders, preset fresh gas flow levels, and real-time display of costs on anesthesia machines) may need to be adapted to the local technology platforms.

## CONCLUSIONS

We believe safe healthcare for pediatric patients is best accomplished by using responsibly sourced materials, resources, and equipment that minimize the impact on the patient’s future environment while attending to their clinical needs. Often, pediatric quality initiatives focus on clinical case management, system-based processes, and guidelines for the individual and local population. Through several clinical practice changes, this QI project addresses the substantial impact our healthcare system has on the pediatric patient’s future environment.
